# The economic burden of asthma in Italy: evaluating the potential impact of different treatments in adult patients with severe eosinophilic asthma

**DOI:** 10.1007/s10198-024-01736-5

**Published:** 2024-12-18

**Authors:** Matteo Scortichini, Francesco Saverio Mennini, Andrea Marcellusi, Martina Paoletti, Carlo Tomino, Paolo Sciattella

**Affiliations:** 1 Economic Evaluation and Health Technology Assessment, Centre for Economic and International Studies, Faculty of Economics, University of Rome Tor Vergata, Rome, Italy; 2 Scientific Direction, IRCCS San Raffaele Roma, Rome, Italy

**Keywords:** Asthma, Severe eosinophilic asthma, Real world evidence, Italy, Economic burden, I11

## Abstract

**Introduction:**

Asthma is a prevalent chronic respiratory condition that significantly impacts public health, with severe asthma subtypes, such as severe eosinophilic asthma, imposing substantial socioeconomic burdens.

**Methods:**

Real-world data from the Italian Health Information System were analyzed to evaluate the economic consequences of asthma in Italy. An in-depth comparative analysis was conducted to investigate the economic implications of various asthma subtypes, focusing on severe eosinophilic asthma. Additionally, the study projected the potential cost-effectiveness of novel treatments aimed at reducing hospitalization rates, specialist visits, and oral corticosteroid use for patients with severe eosinophilic asthma in Italy.

**Results:**

The analysis revealed that severe asthma, and notably severe eosinophilic asthma, places a substantial economic burden on the Italian National Health System. Estimates demonstrated that implementing innovative treatments to mitigate the risks of hospitalization and specialist visits, as well as reducing oral corticosteroid usage in severe eosinophilic asthma patients, could lead to significant cost savings. The cost-consequence analysis indicated potential yearly reductions of €50.0 million (27%) for the treatment of severe asthma and €31.7 million (26%) for severe eosinophilic asthma.

**Conclusions:**

This study presents a comprehensive evaluation of the economic repercussions of severe asthma in Italy. The findings emphasize the necessity of identifying and developing effective therapeutic strategies to improve the management of severe asthma while simultaneously reducing the economic burden on the healthcare system. These results offer valuable insights for healthcare policymakers and practitioners, facilitating evidence-based decisions in asthma management and healthcare policy in Italy.

## Background

Asthma is a chronic disease of the airways affecting around 300 million people across the world [1]. Symptoms are highly heterogeneous, with the most severe forms having an extremely high impact on patients, their families, and society [2]. In Italy recent estimates show an increasing trend of asthma prevalence, from 7.3% in 2014 to 9.1% in 2019, with a higher prevalence in women (9.8%) with respect to men (8.3%) [3]. Severe asthma is characterized by airway inflammation, hyperresponsiveness and airflow limitation, sometime progressing to obstruction [4]. According to the Global Initiative for Asthma (GINA) international recommendations, severe asthma identifies cases of either asthma that is uncontrolled despite high-dose Inhaled Corticosteroids-Long-Acting Beta-Agonists (ICS-LABA) treatment, or asthma that gets worse when high-dose treatment decreases [5]. American Thoracic Society (ATS) and European Respiratory Society (ERS) guidelines define uncontrolled asthma as the disease presenting one of the following conditions: poor symptoms control, frequent severe exacerbations, serious exacerbations (hospitalization) or airflow limitation [6].

The Italian Association of Hospital Allergists and Immunologists (AAIITO) reports the prevalence of severe asthma to be between 5 and 10% of all asthma patients in Italy. In a study conducted on data from the Italian Registry of Severe Asthma (IRSA), asthma was defined uncontrolled in 62.2% of patients with severe asthma [7, 8], while another study from the Italian Severe/uncontrolled Asthma Registry (RItA) reported how asthma was uncontrolled in 39.4% of severe patients [9].

Eosinophilic asthma phenotype is one of the most relevant types of severe asthma. Eosinophils play roles within asthma via the release of inflammatory mediators into tissue sites, causing epithelial damage, airway hyperresponsiveness, mucus hypersecretion and airways remodeling [10–12]. In patients with severe asthma, eosinophilic inflammation can increase the risk of uncontrolled disease, enhanced decline in lung function and a higher risk of mortality [13]. Eosinophilic asthma increases the risk of exacerbations, hospital admissions and the need for higher doses of Oral Corticosteroids (OCS), thus having an impact not only on the patient, but also on the costs sustained by the National Health Service (NHS) [14, 15]. It has also been proven that frequent use of OCS causes side effects both in the short and long term, with the risk of adverse health outcomes being correlated to the treatment dose [16, 17]. A study from the International Severe Asthma Registry (ISAR) on a cohort of severe asthma patients estimated that 82% of the patients had an eosinophilic phenotype, while in Italy several studies found that eosinophilic asthma represents 50–60% of severe asthma cases [18–20].

The aim of this study is to quantify the economic burden of asthma in Italy by implementing a Cost of Illness (CoI) model to estimate direct costs of the disease. Additionally, a Cost-consequence analysis was run on future scenarios data to estimate the potential economic impact of treatments able to reduce the risk of hospitalization, specialistic visits and the use of OCS in patients with eosinophilic asthma.

## Material and methods

### Data sources

This study was conducted using the Health Information System (HIS) for Italy as a whole (60 million inhabitants) and for Local Health Unit (LHU) Umbria 2 (380,000 inhabitants), data were available from 2010 to 2018.

#### Italian HIS

The Italian HIS records all hospital discharges (HD), both ordinary and day-hospital (DH), from public and accredited hospitals. Each record contains, together with a patient specific anonymous code, patient’s demographic (age, sex, residence) and clinical information (primary and up to five secondary diagnoses and procedures, Diagnosis-Related Group – DRG).

#### LHU Umbria 2 HIS

The LHU Umbria 2 HIS routinely collects information on hospitalizations, drug prescriptions, outpatients care and laboratory tests for each patient registered in the Regional Health Care Assistance Registries (approximately 97% of residents). Each patient was identified in the HIS by an anonymous code that allowed deterministic linkage between the databases.

### Methods

#### National analysis

The study population was represented by all residents in Italy hospitalized with a diagnosis of asthma.

Asthma hospitalizations were defined by the presence of Asthma (ICD9CM 493.X) or Eosinophilic asthma (ICD9CM 518.3) in the primary or secondary diagnosis fields. The number of patients, hospitalizations and the average cost by patient were estimated by year. The same estimates were retrieved by considering only hospitalizations within the Major Diagnostic Category “Diseases and Disorders of the Respiratory System” (MDC = 4).

#### LHU Umbria 2 analysis

**Fig. 1:**
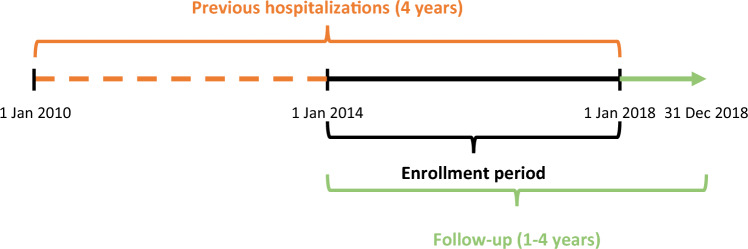
Enrollment scheme for the LHU Umbria2 analysis

A retrospective cohort of subjects with asthma was built by selecting all adult patients (age 18 +) with a hospitalization for asthma (same definition as the one used in the national analysis) during the period 2014–2017 (enrollment period). To select only “incident” cases we excluded all the subjects with a hospitalization for asthma in the period 2010–2013. If a patient had more than a hospitalization during the enrollment period, only the first one was selected as the index event (Fig. 1). All patients were followed for a maximum of 4 years, up until the 31st of December 2018. By linking all the databases available for LHU Umbria 2 it was possible to estimate the total direct costs due to hospitalizations, drugs, and outpatient visits.

**Fig. 2:**
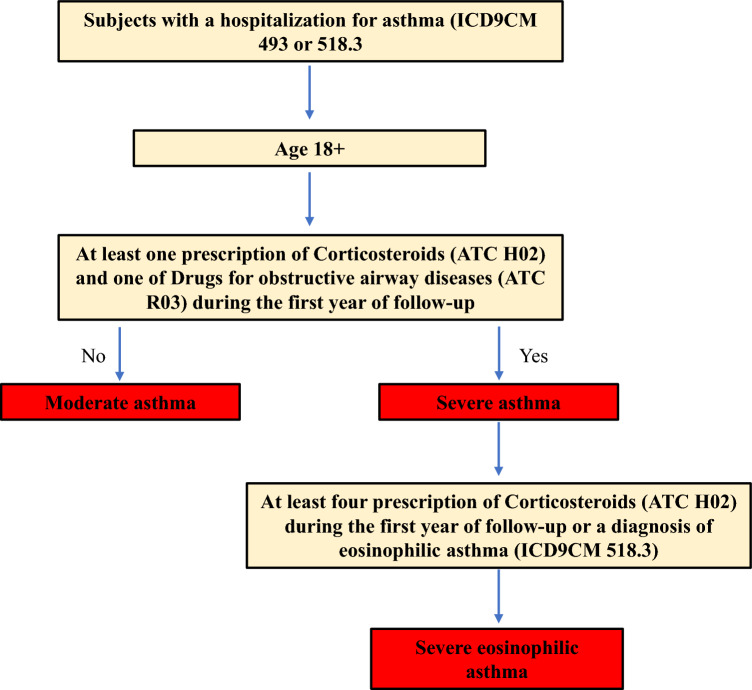
Algorithm for the classification of moderate, severe or eosinophilic asthma patient

Results have been stratified by type of asthma (moderate, severe, eosinophilic). Since the HIS doesn’t report whether a patient has moderate, severe or eosinophilic asthma, in order to classify patients in these categories an algorithm was developed (Fig. 2).

#### Cost-consequence analysis

The distribution of asthma types retrieved from the LHU Umbria 2 analysis was used to extrapolate, at the national level, the number of patients with moderate, severe, and eosinophilic asthma. To take into account the uncertainty associated to the assumption that the distribution of asthma types from LHU Umbria 2 was representative at the national level, the random error of the proportion of patients with severe asthma and eosinophilic asthma was estimated [21]. Then, a bootstrap procedure with 1000 simulations was applied to calculate the 95% confidence interval of the number of patients at the national level [22]. The asthma type-specific average cost estimated in the LHU Umbria 2 analysis was applied to the national estimates to obtain the national expense for asthma.

To estimate the impact of new treatments on the economic burden of severe eosinophilic asthma, estimates obtained from the literature were used to generate future scenarios analyses [23]. The impact of therapeutic innovation on the use of health resources from severe eosinophilic asthma patients is reported in terms of number of hospital admissions, outpatient visits and OCS use before and after treatment.

Specifically, when comparing the 12 months preceding the treatment initiation with the 22 months after, a reduction was observed in terms of asthma related outpatient visits (− 58%), asthma related hospital admissions (−100%) and the median consumption of OCS (mg/day, − 100%) (Table 1). In particular, the reduction in the dosage of OCS was observed in 67% of patients (60% of patients reduced the dose of OCS by 100% and 7% of patients stopped using OCS). The estimating reduction in hospital admissions was applied in the model only to the share of admission classified with MDC “Diseases and Disorders of the Respiratory System”; whereas the reduction in OCS dosage was considered only for patients with OCS prescriptions and only at the percentage of patients with reduction observed in the study. These estimates were applied to the use of resources at the national level in order to obtain the economic impact of new treatments on the costs of severe eosinophilic asthma in Italy.

**Table 1:** Impact of therapeutic innovation on the use of health resources from severe eosinophilic asthma

	Mean outpatient visits per patient (asthma related)	Mean hospital admissions per patient (asthma related)	OCS median consumption (mg/day)
12 months before treatment	2.4	0.2	10
22 months after starting treatment	1.0	0.0	0
% Change	− 58	− 100	−100

## Results

### National analysis

During 2010–2018, on average, every year 33,559 patients in Italy had at least a hospital admission for asthma (36,320 hospital admissions), with an average annual cost per patient of €2549 (Table 2). A major reduction in the number of both patients and hospital admissions has been observed during the study period (− 45.1% and − 45.3%, respectively), while the average cost per patient increased (+ 40.0%). The same trends were observed when considering only hospital admissions with MDC = 4 (Table 2).

**Table 2:** Patients, hospital admission and average cost per patient for asthma Italy 2010–2018

Year	Patients	Hospital admission		Cost per patient	
		Total	MDC = 4	Total	MDC = 4
2010	47,144	51,158	36,952	2081€	1176€
2011	41,246	44,606	31,146	2214€	1179€
2012	37,424	40,405	27,904	2458€	1363€
2013	34,155	36,937	25,243	2621€	1459€
2014	31,559	34,171	23,366	2737€	1546€
2015	29,285	31,596	21,779	2748€	1540€
2016	29,206	31,633	22,441	2804€	1631€
2017	26,143	28,370	19,104	2853€	1590€
2018	25,872	28,006	19,272	2916€	1702€
Mean	33,559	36,320	25,245	2549€	1430€

### LHU Umbria 2 analysis

In 2014–2017, 329 incident cases of asthma were observed, of these 249 (76%) were adults (age category 18 +). 149 subjects (45%) were classified as severe asthma patients. The algorithm assigned 73 out of 149 subjects (22% of the total) to the severe eosinophilic asthma group. On average, for each patient during the follow-up the NHS incurred a cost of €10,112: hospital admissions accounted for 67% of this expense (27% for the index event, 39% for the following ones), followed by drugs (23%) and outpatient visits (11%, Table 3). An adult patient cost on average €12,540, the expense was higher for subjects with severe asthma (€15,359) and twice the overall mean when only patients with severe eosinophilic asthma were considered (€20,065).

**Table 3:** Average cost per patient during the follow-up. LHU Umbria2 2014–2018

	Subjects	Drugs	Outpatient visits	Hospital admissions				Total
				Index hospital admission		Others		
				Total	MDC = 4	Total	MDC = 4	
Total	329	2338 €	1065 €	2747 €	54%	3961 €	21%	10,112 €
Adults	247	3025 €	1289 €	3121 €	47%	5105 €	20%	12,540 €
Severe	149	3940 €	1557 €	3027 €	56%	6835 €	21%	15,359 €
Severe eosinophilic	73	5971 €	2199 €	2988 €	60%	8907 €	24%	20,065 €

The share of cost by type of resource was constant among groups except for the expense for drugs, which raised from 23% in the overall population to 30% in severe eosinophilic patients. The percentage of expense for hospital admissions with MDC = 4 was higher in patients with severe asthma and severe eosinophilic asthma (77% and 84%) with respect to the overall adult population (67%).

### Cost-consequence analysis

In Table 4 the projections of patients at the national level by type of asthma are shown. The analysis reports 12,268 patients with severe asthma every year (95% CI 10,981–13,452), with a total cost of €188.4 million borne by the NHS (95% CI €168.7–€206.6 million). Patients with severe eosinophilic asthma are 6011 (95% CI 4827–7162), accounting for €120.6 million (95% CI €96.9–€143.7 million, Table 4).

**Table 4:** National estimated costs by type of patient

	Subjects	Drugs	Outpatient visits	Hospital admissions		Total
				MDC = 4	Others	
Total	33,559	€ 78,460,942	€ 35,740,335	€ 77,695,461	€ 147,418,311	€ 339,315,049
Adults	20,337	€ 61,519,425	€ 26,214,393	€ 50,595,812	€ 116,696,350	€ 255,025,980
Severe	12,268	€ 48,336,191	€ 19,101,383	€ 38,404,821	€ 82,582,873	€ 188,425,269
Severe eosinophilic	6011	€ 35,888,877	€ 13,217,156	€ 23,624,269	€ 47,870,989	€ 120,601,292

Results from the scenario analysis are reported in Table 5: the implementation of therapeutic innovations would cause a yearly reduction of the NHS expense of €50.0 million (95% CI €44.8–€54.9 million) in the treatment of severe asthma (− 27%). When considering only severe eosinophilic asthma, the reduction would amount to €31.7 million (95% CI €25.4–€37.9), − 26% with respect to the CoI 2018 estimate.

**Table 5:** Cost-consequence analysis results

	Subjects	Drugs	Outpatient visits	Hospital admissions		Total
				MDC = 4	Others	
Severe asthma						
CoI 2018	12,268	€ 48,336,191	€ 19,101,383	€ 38,404,821	€ 82,582,873	€ 188,425,269
Scenario	12,268	€ 47,837,839	€ 7,958,910	€ 0	€ 82,582,873	€ 138,379,622
*Cost-consequence*		***-€ 498,353***	***-€ 11,142,474***	***-€ 38,404,821***	***€ 0***	***-€ 50,045,648***
Severe eosinophilic asthma						
CoI 2018	6011	€ 35,888,877	€ 13,217,156	€ 23,624,269	€ 47,870,989	€ 120,601,292
Scenario	6011	€ 35,483,081	€ 5,507,148	€ 0	€ 47,870,989	€ 88,861,219
*Cost-consequence*		***-€ 405,795***	***-€ 7,710,008***	***-€ 23,624,269***	***€ 0***	***-€ 31,740,073***

Values in bold italics represent the net cost differences between the CoI 2018 and Scenario analyses

The estimate of uncertainty is summarised in Table 6. The scenario analysis shows how the reduction in costs can range from − 24 to − 29% when considering severe asthma patients, and from − 21 to − 31% when considering only patients with severe eosinophilic asthma.

**Table 6:** Uncertainty in national estimates

	Subjects		Total costs		Cost consequence	
	Est	95% CI	Est	95% CI	Est	95% CI
Severe asthma	12,268	10,981–13,452	188,425,269 €	168,657,179 €–206,609,268 €	− 50,045,648 €	− 44,769,365 € to − 54,899,664 €
Severe eosinophilic asthma	6011	4827–7162	120,601,292 €	96,853,755 €–143,705,530 €	− 31,740,073 €	− 25,448,195 € to − 37,856,585 €

## Discussion

The aim of this study was to quantify the economic burden of asthma in Italy by implementing a Cost of Illness (CoI) model to estimate direct costs of the disease. To our knowledge this is the first study using real world data to estimate the economic burden of Asthma in Italy. The SIRIO study in 2007 estimated the economic impact of asthma in Italy on a cohort of patients selected from pneumology centres, with an estimated cost per patient during a 1-year follow-up of €1177 [24]. A study estimating the economic burden of Asthma in the United States from 2008 to 2013 reported an annual per-person incremental medical cost of asthma of $3,266, for a total of 50 billion dollars in medical costs [25]. A Cost-consequence analysis was run on future scenarios data to estimate the potential economic impact of treatments able to reduce the risk of hospitalization, specialistic visits and the use of OCS in patients with severe eosinophilic asthma. A study on a set of Italian patients found that the same treatments caused a 85% reduction in the use of OCS and the annual exacerbation rate going from 5.54 to 0.18 [26]. Another paper found a reduction of OCS dose from 14.8 to 1.49 mg/day [27]. Overall, few studies have focused on projecting asthma related costs borne by the NHS in the future with inconclusive results [28, 29].

Some assumptions and limitations must be acknowledged. While the estimate of asthma prevalence in Italy is based on a national dataset, we made the assumption that LHU Umbria 2 is representative of Italy in terms of therapeutic pathways and distribution of patients: an attempt was made to take into account the uncertainty generated by this assumption, by expressing the number of asthma patients at the national level as a range of potential values. Secondly, the algorithm for the classification of severe and eosinophilic asthma has never been tested before: unfortunately, data on eosinophils count were not available and the diagnosis of eosinophilic asthma in hospital admission has been proven to be highly underestimated in Italy. The analysis protocol and the selection criteria were revised and approved by a team of expert clinicians.

## Conclusion

This study offers a comprehensive characterization of the economic burden of asthma and severe asthma in Italy. The evidence produced in this paper in terms of the efficacy of selected treatments potentially reducing the economic burden of asthma can inform decision makers on future policies so that can make an informed decision based on real world analysis. Cost-Consequence Analysis highlights how having treatments available that, because of their efficacy, are able to reduce hospitalizations, specialistic visits and the use of OCS in patients with severe eosinophilic asthma generates a positive impact both in terms of improving the health of patients with asthma and in terms of reducing direct health care costs.

## Data Availability

The data that support the findings of this study are available from the corresponding author, upon reasonable request.

## References

[1] Global Burden of Disease Collaborative Network: Global Burden of Disease Collaborative Network. Global Burden of Disease Study 2019 (GBD 2019) Results (2019)

[2] Masoli, M., Fabian, D., Holt, S., Beasley, R.: The global burden of asthma: executive summary of the GINA Dissemination Committee Report (2004) 10.1111/j.1398-9995.2004.00526.x 15080825

[3] Cricelli, C., Brignoli, O., Medea, G., Parretti, D., Lombardo, F.P., Aprile, P.L., Lapi, F., Cricelli, I.: Impatto epidemiologico delle cronicità in Medicina Generale

[4] National Asthma Education: Expert Panel Report 3 (EPR-3): guidelines for the diagnosis and management of asthma-summary report 2007. J Allergy Clin Immunol. 120 (2007). 10.1016/j.jaci.2007.09.029 10.1016/j.jaci.2007.09.043 17983880

[5] GINA: Global Initiative for Asthma—GINA What’s new in GINA 2021? Global Strategy for Asthma Management and Prevention. Ginasthma.org (2021)

[6] Chung, K.F., Wenzel, S.E., Brozek, J.L., Bush, A., Castro, M., Sterk, P.J., Adcock, I.M., Bateman, E.D., Bel, E.H., Bleecker, E.R., Boulet, L.P., Brightling, C., Chanez, P., Dahlen, S.E., Djukanovic, R., Frey, U., Gaga, M., Gibson, P., Hamid, Q., Jajour, N.N., Mauad, T., Sorkness, R.L., Teague, W.G.: International ERS/ATS guidelines on definition, evaluation and treatment of severe asthma. Eur. Respir. J. (2014). 10.1183/09031936.00202013 24337046 10.1183/09031936.00202013

[7] Micheletto, C., Bilò, M.B., Antonicelli, L., Bresciani, M., D’amato, G., de Benedictis, E., de Michele, F., Gasparini, S., Giovannini, M., Musarra, A., Vaghi, A.: Severe asthma in adolescents and adults: a national, multicenter registry in real life. Eur. Ann. Allergy Clin. Immunol. (2018). 10.23822/EurAnnACI.1764-1489.69 30039693 10.23822/EurAnnACI.1764-1489.69

[8] Bilò, M.B., Antonicelli, L., Carone, M., de Michele, F., Menzella, F., Musarra, A., Tognella, S., Vaghi, A., Micheletto, C.: Severe asthma management in the era of biologics: insights of the italian registry on severe asthma (irsa). Eur. Ann. Allergy Clin. Immunol. (2021). 10.23822/EurAnnACI.1764-1489.196 33728838 10.23822/EurAnnACI.1764-1489.196

[9] Maio, S., Baldacci, S., Bresciani, M., Simoni, M., Latorre, M., Murgia, N., Spinozzi, F., Braschi, M., Antonicelli, L., Brunetto, B., Iacovacci, P., Roazzi, P., Pini, C., Pata, M., la Grasta, L., Paggiaro, P., Viegi, G., Angino, A., Carrozzi, L., Cerrai, S., di Pede, F., Martini, F., Pala, A.P., Pistelli, F., Sarno, G., Silvi, P., Novelli, F., Ferri, M., Bonifazi, F., Viegi, G.: RItA: The Italian severe/uncontrolled asthma registry. Allergy Eur. J. Allergy Clin. Immunol. (2018). 10.1111/all.13342 10.1111/all.13342 29072882

[10] Nagata, M., Nakagome, K., Soma, T.: Mechanisms of eosinophilic inflammation. Asia Pac. Allergy (2020). 10.5415/apallergy.2020.10.e14 32411579 10.5415/apallergy.2020.10.e14 PMC7203432

[11] Melo, R.C.N., Weller, P.F.: Contemporary understanding of the secretory granules in human eosinophils (2018) 10.1002/JLB.3MR1217-476R PMC6013358 29749658

[12] Busse, W.W., Holgate, S.T.: Asthma and Rhinitis. Blackwell Science, New York (2000)

[13] Price, D., Wilson, A.M., Chisholm, A., Rigazio, A., Burden, A., Thomas, M., King, C.: Predicting frequent asthma exacerbations using blood eosinophil count and other patient data routinely available in clinical practice. J Asthma Allergy (2016). 10.2147/JAA.S97973 26793004 10.2147/JAA.S97973 PMC4708874

[14] de Groot, J.C., ten Brinke, A., Bel, E.H.D.: Management of the patient with eosinophilic asthma: a new era begins. ERJ Open Res. **1**, 00024 (2015) 27730141 10.1183/23120541.00024-2015 PMC5005141

[15] Loftus, P.A., Wise, S.K.: Epidemiology and economic burden of asthma (2015) 10.1002/alr.21547 26010063

[16] Price, D.B., Trudo, F., Voorham, J., Xu, X., Kerkhof, M., Jie, J.L.Z., Tran, T.N.: Adverse outcomes from initiation of systemic corticosteroids for asthma: long-term observational study. J. Asthma Allergy (2018). 10.2147/JAA.S176026 30214247 10.2147/JAA.S176026 PMC6121746

[17] Sullivan, P.W., Ghushchyan, V.H., Globe, G., Schatz, M.: Oral corticosteroid exposure and adverse effects in asthmatic patients. J. Allergy Clin. Immunol. (2018). 10.1016/j.jaci.2017.04.009 28456623 10.1016/j.jaci.2017.04.009

[18] Perez-de-Llano, L., Tran, T.N., Al-ahmad, M., Alacqua, M., Bulathsinhala, L., Busby, J., Canonica, G.W., Carter, V., Chaudhry, I., Christoff, G.C., Cosio, B.G., Costello, R.W., Fitzgerald, J.M., Heaney, L.G., Heffler, E., Iwanaga, T., Jackson, D.J., Kerkhof, M., Rhee, C.K., Menzies-Gow, A., Murray, R., Papadopoulos, N.G., Papaioannou, A.L., Pfeffer, P.E., Popov, T.A., Price, C.A., Sadatsafavi, M., Tohda, Y., Wang, E., Wechsler, M., Zangrilli, J.G., Price, D.B.: Characterization of Eosinophilic and Non-Eosinophilic Severe Asthma Phenotypes and Proportion of Patients with These Phenotypes in the International Severe Asthma Registry (ISAR). Presented at the (2020)

[19] Maio, S., Baldacci, S., Cecchi, L., Viegi, G.: The severe asthma registries: a way to better know and fight the disease. Eur. Ann. Allergy Clin. Immunol. **53**, 99 (2021) 33908224 10.23822/EurAnnACI.1764-1489.203

[20] Heaney, L.G., Perez de Llano, L., Al-Ahmad, M., Backer, V., Busby, J., Canonica, G.W., Christoff, G.C., Cosio, B.G., FitzGerald, J.M., Heffler, E., Iwanaga, T., Jackson, D.J., Menzies-Gow, A.N., Papadopoulos, N.G., Papaioannou, A.I., Pfeffer, P.E., Popov, T.A., Porsbjerg, C.M., Rhee, C.K., Sadatsafavi, M., Tohda, Y., Wang, E., Wechsler, M.E., Alacqua, M., Altraja, A., Bjermer, L., Björnsdóttir, U.S., Bourdin, A., Brusselle, G.G., Buhl, R., Costello, R.W., Hew, M., Koh, M.S., Lehmann, S., Lehtimäki, L., Peters, M., Taillé, C., Taube, C., Tran, T.N., Zangrilli, J., Bulathsinhala, L., Carter, V.A., Chaudhry, I., Eleangovan, N., Hosseini, N., Kerkhof, M., Murray, R.B., Price, C.A., Price, D.B.: Eosinophilic and noneosinophilic asthma: an expert consensus framework to characterize phenotypes in a global real-life severe asthma cohort. In: Chest (2021) 10.1016/j.chest.2021.04.013 33887242

[21] Wilson, E.B.: Probable inference, the law of succession, and statistical inference. J. Am. Stat. Assoc. (1927). 10.2307/2276774

[22] Johnson, R.W.: An introduction to the bootstrap. Teach. Stat. (2001). 10.1111/1467-9639.00050

[23] Vultaggio, A., Aliani, M., Altieri, E., Bracciale, P., Brussino, L., Caiaffa, M.F., Cameli, P., Canonica, G.W., Caruso, C., Centanni, S., D’Amato, M., De Michele, F., Del Giacco, S., Di Marco, F., Menzella, F., Pelaia, G., Rogliani, P., Romagnoli, M., Schino, P., Senna, G., Benci, M., Boarino, S., Schroeder, J.W.: Long-term effectiveness of benralizumab in severe eosinophilic asthma patients treated for 96-weeks: data from the ANANKE study. Respir. Res. **24**, 135 (2023). 10.1186/s12931-023-02439-w 37210543 10.1186/s12931-023-02439-w PMC10200058

[24] Dal Negro, R.W., Micheletto, C., Tosatto, R., Dionisi, M., Turco, P., Donner, C.F.: Costs of asthma in Italy: results of the SIRIO (Social Impact of Respiratory Integrated Outcomes) study. Respir. Med. (2007). 10.1016/j.rmed.2007.07.011 10.1016/j.rmed.2007.07.011 17822890

[25] Nurmagambetov, T., Kuwahara, R., Garbe, P.: The economic burden of asthma in the United States, 2008–2013. Ann. Am. Thorac. Soc. (2018). 10.1513/AnnalsATS.201703-259OC 29323930 10.1513/AnnalsATS.201703-259OC

[26] Vitale, C., Maglio, A., Pelaia, C., D’Amato, M., Ciampo, L., Pelaia, G., Molino, A., Vatrella, A.: Effectiveness of benralizumab in OCS-dependent severe asthma: the impact of 2 years of therapy in a real-life setting. J. Clin. Med. (2023). 10.3390/jcm12030985 36769635 10.3390/jcm12030985 PMC9918073

[27] Sposato, B., Scalese, M., Camiciottoli, G., Carpagnano, G.E., Pelaia, C., Santus, P., Pelaia, G., Palmiero, G., Tomassi, M.D.I., Ronchi, M.C., Cameli, P., Bargagli, E., Ciambellotti, L., Rizzello, S., Sglavo, R., Coppola, A., Lacerenza, L.G., Gabriele, M., Radovanovic, D., Perrella, A., Ricci, A., Rogliani, P.: Severe asthma and long-term Benralizumab effectiveness in real-life. Eur. Rev. Med. Pharmacol. Sci. (2022). 10.26355/eurrev_202210_30016 36314316 10.26355/eurrev_202210_30016

[28] Azzano, P., Dufresne, É., Poder, T., Bégin, P.: Economic considerations on the usage of biologics in the allergy clinic. Allergy **76**, 191–209 (2021) 32656802 10.1111/all.14494

[29] Reibman, J., Tan, L., Ambrose, C., Chung, Y., Desai, P., Llanos, J.P., Moynihan, M., Tkacz, J.: Clinical and economic burden of severe asthma among US patients treated with biologic therapies. Ann. Allergy Asthma Immunol.Immunol. **127**, 4589 (2021) 10.1016/j.anai.2021.03.015 33775904

